# Isolation, Characterization and Performance of Autochthonous Spray Dried Lactic Acid Bacteria in Maize Micro and Bucket-Silos

**DOI:** 10.3389/fmicb.2018.02861

**Published:** 2018-11-28

**Authors:** Patricia Burns, María F. Borgo, Ana Binetti, Melisa Puntillo, Carina Bergamini, Roxana Páez, Rodolfo Mazzoni, Jorge Reinheimer, Gabriel Vinderola

**Affiliations:** ^1^Instituto de Lactología Industrial (INLAIN, UNL–CONICET), Facultad de Ingeniería Química, Universidad Nacional del Litoral, Santa Fe, Argentina; ^2^Instituto Nacional de Tecnología Agropecuaria (INTA EEA Rafaela), Rafaela, Argentina; ^3^Fragaria SRL, Villa Cañas, Argentina

**Keywords:** maize, silage, lactic acid bacteria, spray-drying, inoculant, aerobic stability

## Abstract

The aim of this study was to isolate, identify and characterize lactic acid bacteria (LAB) from spontaneously fermented maize silage, and evaluate their performance as spray-dried (SD) cultures to enhance the fermentation and the aerobic stability of maize micro-silos. Eleven strains of LAB were characterized for growth kinetics, the capability to grow in vegetable-based medium (VBM), production of organic acids and the ability to tolerate heat–stress. Three strains (*Lactobacillus plantarum* Ls71, *Pediococcus acidilactici* Ls72, and *Lactobacillus buchneri* Ls141) were selected and further characterized for the ability to grow as single strain or in co-culture in MRS and VMB medium, to survive at freeze and spray-drying process, for their performance as SD bacteria in micro-silos and for the aerobic stability in bucket silos. *L. buchneri* Ls141 showed the highest growth capability in VBM and produced the highest amount of acetic acid, while *L. plantarum* Ls71 produced the highest amounts of lactic acid. *P. acidilactici* Ls72 was the most heat-resistant strain, with a reduction of 0.2 log_10_ CFU/mL (15 min at 55°C). The three strains satisfactorily tolerated both spray and freeze-drying. After 4 days of fermentation, all the samples reached a pH value of about 3.7–3.8. A significantly lower cell load of filamentous fungi and yeasts (< 3 log_10_ CFU/g) and a higher concentration of total LAB (> 8.7 log_10_ CFU/g) was observed after 30 days of fermentation. A greater amount of acetic acid, crude protein, ash and ammonia nitrogen/total nitrogen was detected in inoculated silages. A significant reduction of filamentous fungi and yeasts was also observed in inoculated bucket silos after 50 d of fermentation. The aerobic stability was significantly improved in inoculated silage since the temperature remained stable after 16 days (384 h). On the contrary, an increase of 5°C was observed in control samples after 1 day. The selected strains have the potential to be produced as SD silage inoculant as they were able to accelerate the fermentation process, to control filamentous fungi and yeasts, to improve some nutritional and chemical parameters of silage and to improve aerobic stability.

## Introduction

Ensiling is a crop preservation method based onnatural lactic acid fermentation under anaerobic conditions. Epiphytic LAB plays a major role in silage fermentation; however, their count is usually variable in silage crops (Lin et al., 1992). The use of different chemical and biological additives is suggested to improved silage quality (Ávila et al., 2014). According to the composition 6 categories of additives can be mentioned: homofermentative LAB, obligate heterofermentative LAB, combination inoculants (containing obligate heterofermentative + homofermentative LAB), other inoculants (non LAB), chemicals, and enzymes (Muck et al., 2018). Microbial inoculants composed of strains of facultatively homofermentative LAB such as *L. plantarum*, *L. casei*, various *Pediococcus* species and *Enterococcus faecium* are the older and most common commercially available additives. In the late 1990s, a new class of inoculants appeared in the market, based on obligate heterofermentative LAB such as *L. buchneri*. This bacterium grows slowly even after the active fermentation period is finished, producing acetic acid that can inhibit the growth of yeasts and filamentous fungi, improving aerobic stability (Kleinschmit and Kung, 2006; Ávila et al., 2014; Muck et al., 2018).

Nowadays, there is a growing interest in the use of microbiological additives to improve the fermentation process, thus resulting in better quality silage. In Argentina, most of the inoculants are provided by foreign companies as freeze-dried cultures. Spray drying is an interesting and promising low-cost alternative to freeze-drying and allows the continuous production of large amounts of dried cells within short time periods. During this process, bacterial cultures are exposed to different stresses due to the quite harsh conditions of temperature required for product dehydration, which can cause a partial thermal inactivation of cells. It was observed that the success of its application is highly strain specific (Paéz et al., 2012, 2013).

In this context, the aim of this study was to isolate and characterize LAB from spontaneously fermented maize silage, formulate a spray-dried (SD) inoculant and assess its performance to improve the fermentation profile and the aerobic stability of maize micro-silos.

## Materials and Methods

### Samples and Isolation of LAB

Spontaneously fermented maize silages samples (Zea mays) were obtained from two local farms (from Esperanza and Recreo cities, located at 35 and 20 km, respectively, from Santa Fe, Argentina) at the beginning (time = 0) and after 7, 14, and 21 days of silage-making. Samples were taken immediately to the lab for microbiological analysis. Each sample (10 g) was mixed with 90 mL of peptone water (0.1% w/v, Britannia, Buenos Aires, Argentina), homogenized (stomacher, 120 s, low speed) serially diluted and surface-plated on MRS (de Man, Rogose, and Sharpe), MRS bile, MRS-LP, and M17 agar (Biokar, Beauvais, France) (Vinderola and Reinheimer, 1999). Plates were incubated aerobically (or anaerobically, MRS-LP) at 34°C for 72 h (Oxoid, Basingstoke, United Kingdom). Twenty two colonies presenting typical LAB morphology (examined by phase-contrast microscopy, 1000×) were isolated and purified for Gram-staining reaction, mobility, catalase activity and gas production (in MRS broth with Dürham tube). Presumptive LAB isolates were frozen stored in MRS broth added with 20% v/v glycerol (Ciccarelli, Buenos Aires, Argentina) at -20°C and -70°C.

### Identification of Isolates

Total DNA of isolates was obtained from overnight cultures (20 h) by using the GenElute^TM^ Bacterial Genomic DNA kit (Sigma, St. Louis, MO, United States) according to the manufacturer’s instructions. Purified DNA samples were stored at -20°C until use. The identity of isolates was analyzed by amplifying (primers pA: AGA GTT TGA TCC TGG CTC AG, and pH: AAG GAG GTG ATC CAG CCG CA), sequencing and comparing a 1500 bp fragment within their 16S rRNA gene (Edwards et al., 1989). All PCR reactions were performed using 2 μL of diluted (1:50) DNA as template, 2.5 U Taq DNA polymerase (GE Healthcare, Little Chalfont, United Kingdom), 200 μM dNTPs (GE Healthcare) and 100 nM each primer (Sigma-Genosys, The Woodlands, TX, United States) in a final volume of 50 μL. Amplifications were performed in a GeneAmp PCR System (Applied Biosystems, Foster City, CA, United States) under the following conditions: 3 min at 94°C, 36 cycles of 1 min at 94°C, 2 min at 51°C and 2 min at 72°C, and a final step of 7 min at 72°C. The PCR products were separated on 0.8% (w/v) agarose gels in TBE buffer, stained with GelRed (Biotium, Hayward, CA, United States) and visualized under UV light (Sambrook and Russell, 2001). Amplicons were purified with MicroSpin Columns (GE Healthcare) and their nucleotide sequences were determined by primer extension at the DNA Sequencing Service of Macrogen (Seoul, Korea). The identity of isolates was checked by nucleotide-nucleotide BLAST of the NCBI database^1^.

### Growth Kinetics

MRS broth was inoculated (1% v/v) with an overnight culture of each strain [(previously washed twice with PBS (phosphate buffer solution)] and incubated at 30, 34, 37, and 43°C in a 96-well microplate (Thermo Scientific Multiskan FC Microplate Photometer). Optical density (OD_570 nm_) was measured every 30 min during 24 h. The OD data were modeled with Statistica software (Version 8.0; StatSoft., Tulsa, OK, United States), by using the Gompertz equation as modified by Zwietering et al. (1990), in order to evaluate the cell growth parameters [(μ_max_: maximum specific growth rate as variation of OD_570 nm/h_; λ: lag time in hours)].

At the same time, MRS broth (10 mL) was inoculated (1% v/v) and incubated for 24 h (at 30, 34, 37, and 43°C). Colony counts (MRS agar, 34°C, 72 h, aerobiosis) were carried out at the beginning and after 24 h of incubation. Results are expressed as (Δ log_10_ CFU/mL), where Δ is the difference between cell counts after 24 h and the initial count.

### Growth Kinetics in Vegetable-Based Medium and Determination of Organic Acids and Carbohydrates

#### Vegetable-Based Medium (VBM)

A fresh (non-fermented) sample of chopped maize obtained before silage fermentation was mixed (1:10) with distilled water, homogenized (stomacher, 120 s, low power), filtered (QUANTY JP41 Faixa Preta, Londrina, PR, Brazil) and centrifuged (5000 × *g*, 10 min, 5°C). The pH of the supernatant was adjusted to 6.5 with 1 M NaOH, aliquoted and stored at -20°C. Before each experiment, the medium was autoclaved (121°C, 15 min).

#### Cell Growth in VBM and Determination of Organic Acids and Carbohydrates by HPLC

An overnight fresh culture of each strain (previously washed twice with PBS) was inoculated (1% v/v) in VBM and anaerobically incubated at 34°C, during 72 h. Colony counts were performed at 0, 24, 48, and 72 h in MRS agar. Results are expressed as (Δ log_10_ CFU/mL), where Δ is the difference between cell counts after 24, 48, and 72 h and the initial count. pH was measured after 72 h of culture (Orion 3 Star, Thermo Scientific, Beverly, MA, United States) and the supernatant was recovered by centrifugation (8000 × *g*, 15 min, 5°C). The quantification of organic acids (lactic, acetic, and propionic) and carbohydrates (glucose and fructose) was performed by HPLC according to Vénica et al. (2014). Chromatographic separation was carried out isocratically at 65°C with a mobile phase of 10 mM H_2_SO_4_ at a flow rate of 0.6 mL/min on an Aminex HPX-87H column (300 × 7.8 mm) equipped with a cation H+ microguard cartridge (Bio-Rad Laboratories, United States). The supernatant of cultures after centrifugation was diluted 1:3 with 10 mM H_2_SO_4_, filtered through 0.45 μm membrane (Millex, Millipore, Brazil) and injected into the chromatograph, using a loop of 60 μL. HPLC equipment consisted of a quaternary pump, an on-line degasser, a column oven, a UV-visible detector (all Series 200) and a refractive index detector thermostatized at 35°C (Series Flexar) (Perkin Elmer, United States). The UV detector was set at 210 nm for the detection of organic acids, while the IR detector setting at 35°C was used for the analyses of carbohydrates. Data were collected and processed on a computer with the software Chromera^®^ (Perkin Elmer).

### Tolerance to Heat Stress

Strains were grown overnight in MRS broth, centrifuged (8000 × *g*, 15 min, 5°C), washed twice in PBS (pH 7.2) and resuspended in 5 mL of the same buffer. Cell suspensions were aliquoted (1 mL) and placed into a water bath at 55°C with mild stirring. At time = 0 and after 5 and 15 min, samples were removed and placed in a cold-water bath. Colony counts (MRS agar, 34°C, 72 h, aerobiosis) were carried out to determine the tolerance of strains to heat stress.

### Microbial Growth as Single or Co-culture in MRS and VBM

Fresh cultures of the selected strains (*Lactobacillus plantarum* Ls71, *Pediococcus acidilactici* Ls72 and *Lactobacillus buchneri* Ls141, see discussion section) in MRS broth were centrifuged (5000 × *g*, 20 min, 8°C), washed twice with sterile PBS and resuspended in the same buffer to obtain a cell suspension of *c.a.* 2 × 10^8^ CFU/mL. MRS broth or VBM was inoculated (1% v/v) with the strains as pure cultures (positive control of growth) or in co-culture (2 or 3 strains together) and anaerobically incubated at 34°C during 24 h. Cell counts (MRS agar, 34°C, 72 h, aerobiosis) were performed at the beginning and after 24 h. It was previously checked that strains could be differentiated (MRS agar surface plating) according to their colony morphology.

### Resistance to Spray and Freeze-Drying

Overnight cultures in MRS broth of the selected strains (*L. plantarum* Ls71, *P. acidilactici* Ls72, and *L. buchneri* Ls141) were centrifuged (5000 × *g*, 20 min, 8°C), washed twice with PBS (pH 7.2) and resuspended in 20% (w/v) (1:1) maltodextrin (Gelfix, Buenos Aires, Argentina)-whey protein concentrate (WPC80) (Arla Foods, Porteña, Córdoba, Argentina).

Cell suspensions (prepared as described above) were SD in a laboratory scale spray dryer (Buchi mini spray dryer model B290, Flawil, Switzerland). An inlet air temperature of 138–145°C, an outlet temperature of 81–83 °C and a flux of 600 L/h were used. Cell suspensions were atomized and sprayed into the drying chamber using a two-fluid nozzle. The product dried almost instantaneously, and the residence time was negligible. Three independent replicates were performed for each strain. Cell counts, before and after spray drying, were performed in MRS agar (72 h, 34°C, aerobiosis).

Cell suspensions (prepared as described above) were freeze-dried in a laboratory scale freeze dryer (Christ Alpha 1–4 LD Plus, Osterode am Harz, Germany). Freeze drying conditions were 0.002 mBar, -55°C, 20 h. Three independent replicates were performed for each strain. Cell counts, before and after freeze drying, were performed in MRS agar (72 h, 34°C, aerobiosis).

### Evaluation of the Selected Spray Dried Bacteria as Inoculants in Experimental Maize Silage

Silage was made with fresh-cut maize using a small-scale system of silage fermentation (Cai et al., 1998; Johnson et al., 2005). Chopped maize was taken immediately to the lab for silage-making. Spray dried strains (*L. plantarum* Ls71, *P. acidilactici* Ls72, and *L. buchneri* Ls141) were prepared as described above. The silage treatments were designed as follows: (i) untreated control (UC) (sprayed with sterile water); (ii) substrate (S) (sprayed with a suspension of 20% (w/v) maltodextrin-WPC); (iii) enzyme (E) (sprayed with 0.05% (w/v) *Acremonium* fungal cellulase solution (Milar Enzimas S.R.L., Buenos Aires, Argentina); (iv) spray dried bacteria (SDB) [(SD culture resuspended in sterile water and sprayed on the forage to a final concentration (of each strain) of *c.a.* 5 × 10^6^ CFU/g of cropped maize)], and (v) spray dried bacteria + enzyme (SDB-E). All treatments were applied at a rate of 20 mL/kg of the corresponding solution. Approximately 400 g portions of each treatment (in triplicate, for each sampling day) were vacuum-packaged (Turbovac, Bosch) in 200 × 450 mm, 58 microns, high barrier shrink bags (Cryovac: BC40LA) OTR (Oxygen Transmission Rate: 10–18 [(cm^3^/m^2^) × 24 h × bar]). The micro-silos were stored at room temperature (25°C) for 60 days. pH was measured after 0, 4, 7, 30, and 60 days of fermentation. Ten grams of each sample were added with 90 mL of sterile distilled water and homogenized (stomacher, 120 s, low power). Microbiological analyses and quantification of organic acids and carbohydrates by HPLC (Vénica et al., 2014) were carried out at the beginning and after 30 and 60 days of storage. Total LAB were enumerated in MRS agar (34°C, 48 h, aerobiosis) and yeasts and filamentous fungi in chloramphenicol glucose agar (Biokar, Beauvais, France) (25°C, 7 d, aerobiosis). Chemical analyses were performed after 30 [Dry Matter (g/kg DM; PROMEFA-v2 AOAC, 1990 N° 130.15 and N° 167.03), pH, Ammonia Nitrogen/Total Nitrogen (N_NH3_/N_T_; Blain and Urtinett, 1954) and Crude Protein (g/kg CP; AOAC, 1998 N° 976.05)] and 60 days of fermentation [DM, pH, N_NH3_/N_T_, CP, Ash (g/kg), Acid Detergent Insoluble Nitrogen/Total Nitrogen (NADIN/NT). Acid detergent fiber in feeds. Filter bag technique, Ankom200), Acid Detergent Fiber (g/kg ADF; ANKOM Method validated with ISO13906:2008), Neutral Detergent Fiber (g/kg NDF; ANKOM Method validated with ISO16472:2006), and Metabolisable Energy (ME; Mcal/kg DM)] (Laboratorio de Análisis de Forrajes, Concentrados e Insumos Agropecuarios de la Facultad de Ciencias Agrarias (FCA), UNL, Esperanza, Santa Fe, Argentina). Analyses were carried out in triplicate.

### Bucket Silos and Aerobic Stability Measurement

Chopped maize was taken immediately to the lab for silage-making. Spray dried strains (*L. plantarum* Ls71, *P. acidilactici* Ls72, and *L. buchneri* Ls141) were prepared as described in 2.7. For these assay two groups were used: UC and SDB-E. Both treatments were prepared as described above and bacteria were sprayed to a final concentration of 5 × 10^6^ CFU/g of cropped maize. Twelve kilograms bucked silos were prepared (in triplicate), compacted, sealed and stored at room temperature (25°C) during 50 days. At the end of the fermentation period, pH and cell counts of total LAB and yeast and filamentous fungi were determined.

Two kilograms of well-mixed silage from the medium portion of each bucket silo (free of visible spoilage) were placed back (without packing) into clean plastic bags (2 bags for each silo, placed inside an expanded polystyrene box covered with a cloth). A digital indoor-outdoor thermometer (Boeco, Hamburg, Germany) was inserted into the center of each silage mass and the temperature was recorded four times a day. The aerobic stability was defined as the time taken to increase the temperature of the feed by 2°C above the ambient temperature (Reich and Kung, 2010).

### Statistical Analysis

Data were analyzed using the one-way ANOVA procedure of SPSS software (SPSS Inc., Chicago, IL, United States). The differences between means were detected by Tukey and Duncan’s Multiple Range test. Data were considered significantly different when *P* < 0.05.

## Results

### Isolation and Identification of LAB

Out of 22 isolates, 11 presumptive LAB were subjected to genetic identification by sequencing the 16S region of the rDNA, resulting in *Lactobacillus plantarum* (Ls71 and Hv75); *Lactobacillus amylovorus* (Hv142, Hv212, and Hv214); *Lactobacillus panis* (Hv71, Hv73, and Hv77); *Lactobacillus fermentum* Hv76, *Lactobacillus buchneri* Ls141, and *Pediococcus acidilactici* Ls72. *Lactobacillus* strains were originally isolated from MRS agar and *P. acidilactici* Ls72 from M17 agar. Silage origin of the strains is shown in Table 1.

**Table 1:** Identification, description of the kinetic growth parameters (μ_max_ and λ) and optimal growth temperatures of LAB isolated from silages at different times of fermentation.

Isolate N°	Silage origin	Day of isolation	Identification	Strain name	μ_max_			λ			Optimal growth temperature (°C)
							
					30°C	34°C	37°C	30°C	34°C	37°C	
1	Recreo	7	*L. plantarum*	Ls71	0.31	0.27	0.27	3.25	2.83	2.68	34–37
2	Recreo	7	*P. acidilactici*	Ls 72	0.22	0.13	0.17	3.38	3.08	2.54	34–37
3	Recreo	14	*L. buchneri*	Ls141	0.10	0.08	0.06	15.50	13.00	14.10	34–37
4	Esperanza	7	*L. plantarum*	Hv75	0.31	0.35	0.33	3.12	4.47	2.98	34–37
5	Esperanza	7	*L. fermentum*	Hv76	0.13	0.19	0.17	2.74	2.08	1.37	34–37
6	Esperanza	21	*L. amylovorus*	Hv214	0.07	0.15	0.23	12.7	6.09	5.48	37
7	Esperanza	21	*L. amylovorus*	Hv212	0.11	0.15	0.19	7.52	4.91	5.34	34–37
8	Esperanza	14	*L. amylovorus*	Hv142	0.15	0.16	0.21	5.96	3.25	2.32	37
9	Esperanza	7	*L. panis*	Hv73	nd	nd	nd	nd	nd	nd	nd
10	Esperanza	7	*L. panis*	Hv71	nd	nd	nd	nd	nd	nd	nd
11	Esperanza	7	*L. panis*	Hv77	nd	nd	nd	nd	nd	nd	nd


**μ*_*max*_, the maximum specific growth rate as variation of O.D_*560nm/h.*_; *λ*, lag time in hours. Nd, not detected.*

### Growth Kinetics

**FIGURE 1:**
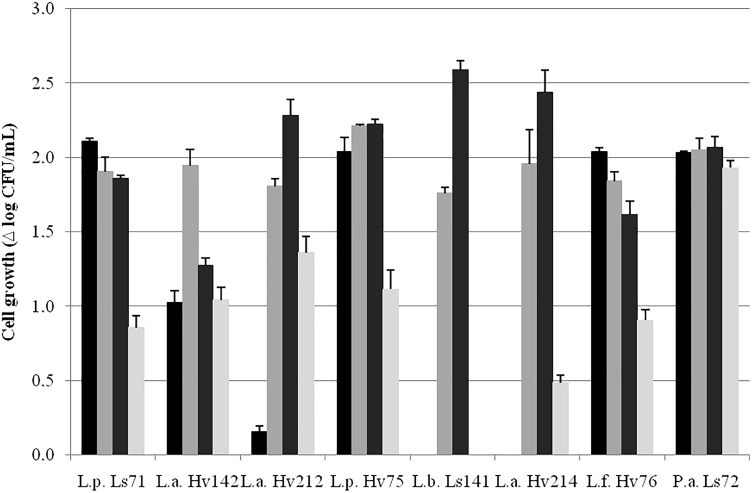
Cell growth in MRS broth (Δ log_10_ CFU/mL ± SEM) at 30 (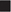), 34 (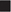), 37 (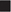), and 43 (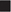) °C after 24 h, aerobiosis.

Cell growth parameters (μ_max_ and λ) at 30, 34, and 37°C are shown in Table 1. Since only *P. acidilactici* Ls72 showed a fast growth kinetic at 43°C, the data, at this temperature, were not modeled. For all the strains, except for *L. panis* Hv71, Hv73, and Hv77 since they presented poor growth capacity after several transfers in MRS broth, the most suitable growth temperatures were 34 and 37°C (Table 1). *L. plantarum* Ls71 and *L. fermentum* Hv76 showed the shortest lag phase both at 34°C and 37°C while *L. buchneri* Ls141 showed a long exponential phase until almost 13–14 h of culture. At 30°C, almost all the strains had a longer λ. The cell growth (Δ log_10_ CFU/mL) after 24 h at the different temperatures is shown in Figure 1. *P. acidilactici* Ls72 was the only strain able to grow at the four temperatures assessed. *L. buchneri* Ls141 was not able to grow at 30 or 43°C. The results indicate that most of the isolates are mesophilic LAB. For further studies, a growth temperature of 34°C was chosen.

### Growth Kinetics in Vegetable-Based Medium and Determination of Organic Acids and Carbohydrates by HPLC

**FIGURE 2:**
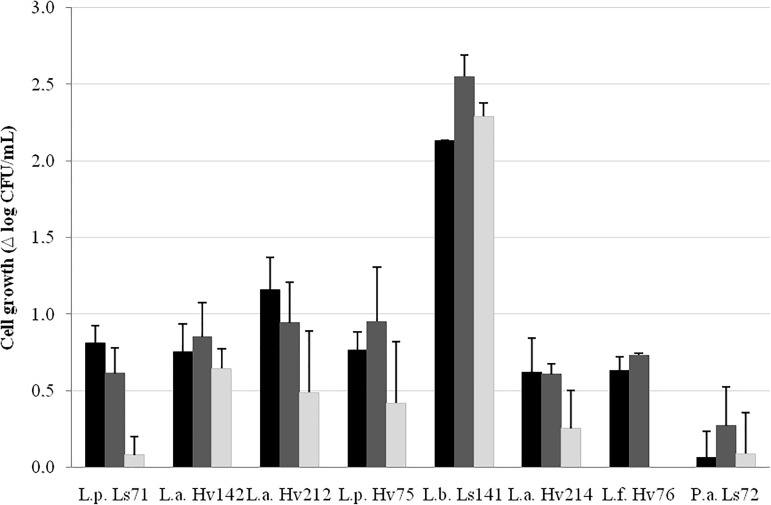
Cell growth (Δ log_10_ CFU/mL ± SEM) in VBM after 24 (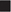), 48 (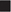), and 72 h (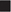) at 34°C, aerobiosis.

The capacity to grow in the VBM was strain-dependent (Figure 2). *L. buchneri* Ls141 showed the highest growth capacity (> 2 log_10_ CFU/mL after 24 h of incubation) while *P. acidilactici* Ls72 was able to grow only 0.3 log_10_ CFU/mL (after 48 h). Most of the strains grew between 0.6 and 1.1 log_10_ CFU/mL after 24 h of incubation and then viability loss was observed, except for *L. buchneri* Ls141 that showed a maximum growth at 48 h.

The determination of organic acids and carbohydrates was performed after 72 h of fermentation (Table 2). Two peaks with retention time similar to glucose and fructose were detected in the chromatograms with IR detector. It is important to highlight that sucrose is hydrolyzed during the analysis due to the chromatography conditions used (high temperature and low pH) (Vénica et al., 2014). So, the results of glucose and fructose are the sum of the basal level in the maize and that providing from sucrose hydrolysis, if it were present. Propionic acid was not detected in any of the samples. *L. plantarum* Ls71 and *L. plantarum* Hv75 produced the highest amounts of lactic acid which correlated with the lowest pH values and an increased consumption of carbohydrates (concentration below the detection limit). *L. buchneri* Ls141 and *L. fermentum* Hv76 produced the highest amounts of acetic acid and the lowest concentrations of lactic acid. pH values were above 4 and there was less consumption of carbohydrates. *L. amylovorus* Hv212, Hv214, and Hv142 acidified the VBM (pH ≈3.8), no acetic acid was detected, and the concentration of lactic acid was between 1.1 and 1.5 mg/mL.

**Table 2:** pH values and concentration of organic acids, glucose and fructose (mg/mL ± SD) in vegetable-based medium (VBM) after 72 h at 34°C, anaerobiosis.

Strains	pH	Concentration (mg/mL)			
		
		Lactic acid	Acetic acid^∗^	Glucose^#^	Fructose^#^
L.b. Ls141	4.33 ± 0.04	0.82 ± 0.01	0.16 ± 0.05	0.20 ± 0.03	0.53 ± 0.04
L.p. Hv75	3.66 ± 0.04	2.41 ± 0.15	0.15 ± 0.04	nd	nd
L.p. Ls71	3.65 ± 0.06	2.35 ± 0.05	0.09 ± 0.04	nd	nd
L.a. Hv142	3.76 ± 0.06	1.51 ± 0.07	nd	0.27 ± 0.04	0.51 ± 0.02
L.f. Hv76	4.77 ± 0.11	0.59 ± 0.08	0.18 ± 0.01	0.42 ± 0.08	0.48 ± 0.02
L.a. Hv214	3.88 ± 0.01	1.21 ± 0.01	nd	0.36 ± 0.07	0.60 ± 0.08
L.a. Hv212	3.88 ± 0.10	1.14 ± 0.15	nd	0.31 ± 0.02	0.65 ± 0.08
P.a. Ls72	4.23 ± 0.10	1.01 ± 0.09	nd	0.41 ± 0.09	0.93 ± 0.01
VBM medium	6.5	nd	nd	1.00	1.18


*nd, not detected. ^∗^Detection limit: 0.05 mg/mL; ^#^detection limit: 0.10 mg/mL.*

### Tolerance to Heat Stress

**FIGURE 3:**
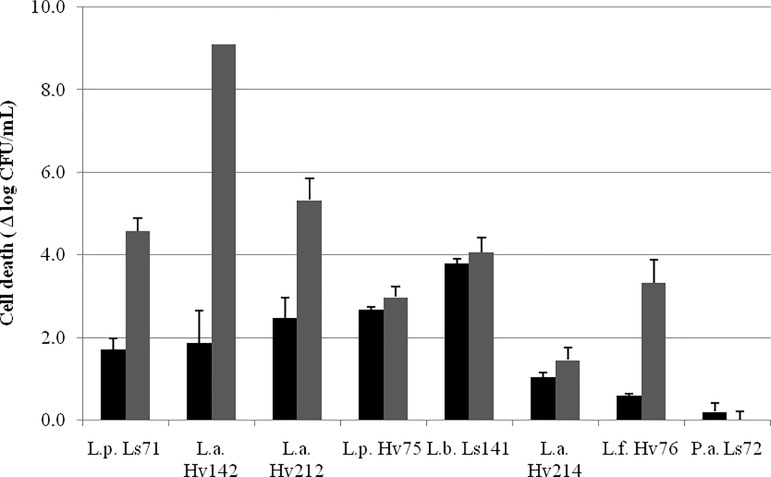
Cell death (Δ log_10_ CFU/mL ± SEM) after 5 (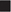) and 15(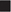) min at 55°C.

Heat-resistance results (cell death after 5 and 15 min at 55°C) are shown in Figure 3. Heat tolerance was strain dependent. *P. acidilactici* Ls72 was the most resistant strain, with a reduction in cell viability of 0.2 log_10_ CFU/mL both after 5 and 15 min. After 5 min there was a cell death between 0.2 and 3.8 log_10_ CFU/mL for all the strains. It was expected that strains with higher thermal resistance will better resist dehydration by spray drying.

### Microbial Growth as Single or Co-culture in MRS and VBM

For both MRS and VBM the initial inoculum of all the strains was about 2 × 10^6^ CFU/mL. Results are shown in Table 3. As pure culture, all the strains were able to grow more than 3 log_10_ CFU/mL in MRS broth. When the strains were co-cultured a cell growth of more than 2.1 log_10_ CFU/mL was observed for all of them. When VBM was used, a cell growth between 1.16 and 1.89 log_10_ CFU/mL was observed. The combination *L. plantarum* Ls71/*P. acidilactici* Ls72 and *L. plantarum* Ls71/*L. buchneri* Ls141 showed a cell growth of about 1 log_10_ CFU/mL but when *P. acidilactici* Ls72 was cultured with *L. buchneri* Ls141 (or the 3 strains together) it was not able to grow.

**Table 3:** Cell growth of strains (Δ log_10_ CFU/mL ± SD) as single or co-cultured in MRS and VBM after 24 h at 34°C, aerobiosis.

Strain/combination	Cell growth (Δ log_10_ CFU/mL)					
	
	MRS			VBM		
L.p. Ls71	3.14 ± 0.30^∗^			1.89 ± 0.33		
P.a. Ls72	3.11 ± 0.16^∗^			1.16 ± 0.35		
L.b. Ls141	3.04 ± 0.02^∗^			1.62 ± 0.77		

	**Ls71**	**Ls72**	**Ls141**	**Ls71**	**Ls72**	**Ls141**
		
Ls71 Ls72	2.95 ± 0.07	2.78 ± 0.21	–	1.26 ± 0.07	1.26 ± 0.20	–
Ls71/Ls141	3.28 ± 0.20	–	2.35 ± 0.03	1.24 ± 0.29	–	0.93 ± 0.04
Ls141/Ls72	–	3.03 ± 0.42	2.15 ± 0.83	–	0.19 ± 0.27	1.12 ± 0.37
Ls71/Ls72/Ls141	2.82 ± 0.25	2.80 ± 0.13	2.47 ± 0.46	1.09 ± 0.20	0.52 ± 0.01	1.31 ± 0.35


***^∗^**The cell growth of each stain cultured in MRS differs significantly with respect to VBM (*p* < 0.05).*

### Resistance to Spray and Freeze-Drying

All the strains exhibited satisfactory resistance to both dehydration processes. After freeze-drying, a cell death of 0.56 ± 0.37, 0.46 ± 0.24, and 0.33 ± 0.38 log_10_ CFU/mL was observed for *L. buchneri* Ls14, *L. plantarum* Ls71, and *P. acidilactici* Ls72, respectively. *L. plantarum* Ls71 was the most sensitive strain to spray dried with a loss of viability of 0.92 ± 0.37 log_10_ CFU/mL. On the contrary, *P. acidilactici* Ls72 was the most resistant strain with a cell death of 0.02 ± 0.19 log_10_ CFU/mL (viability rate 95 ± 9%).

### Evaluation of Selected Spray Dried Cultures as Inoculants in Experimental Maize Silage

pH values of micro-silos after 0, 4, 7, 30, and 60 days of fermentation at 25°C are shown in Table 4. After 4 days of fermentation, all the samples reached a pH between 3.73 and 3.86 and these values remained stable until the end of the fermentation period.

**Table 4:** pH values of micro-silos (mean ± SD) after 0, 4, 7, 30, and 60 days of fermentation at 25°C.

Sample	Days of ensiling				
	
	0	4	7	30	60
UC	5.90 ± 0.09	3.84 ± 0.01	3.81 ± 0.01	3.78 ± 0.00	3.75 ± 0.01
S	5.90 ± 0.09	3.86 ± 0.06	3.84 ± 0.01	3.79 ± 001	3.80 ± 0.02
E	5.90 ± 0.09	3.84 ± 0.03	3.75 ± 0.01	3.73 ± 0.02	3.78 ± 0.07
SDB	5.90 ± 0.09	3.74 ± 0.00^∗^	3.72 ± 0.02	3.80 ± 0.03	3.90 ± 0.08
SDB+E	5.90 ± 0.09	3.73 ± 0.02^∗^	3.71 ± 0.03	3.73 ± 0.02	3.73 ± 0.03


*^∗^Values within the same time differ significantly with respect to UC group (*p* < 0.05). UC, untreated control; S, substrate; E, enzyme; SDB, spray dried bacteria; SDB-E, spray dried bacteria + enzyme.*

Microbiological analyses are shown in Table 5. After 30 days of fermentation there was a significantly lower (*p* < 0.05) cell load of filamentous fungi and yeasts ( < 3 log_10_ CFU/g) for both inoculated micro-silos (SDB and SDB-E), compared to UC, S, and E samples. Likewise, the number of LAB was significantly higher for inoculated samples (more than 8.7 log_10_ CFU/g) and about 6 log_10_ CFU/g for non-inoculated ones. After 60 days of fermentation neither filamentous fungi nor yeasts were detected in SDB and SDB-E (< 1 log_10_ CFU/g) micro-silos, whereas counts higher than 5.3 log_10_ CFU/g were found for UC, S, and E micro-silos.

**Table 5:** Microbiological analyses (log_10_ CFU/g ± SD) of micro-silos during storage at 25°C.

Sample	Cell count (log_10_ CFU/g ± SD)					
	
	*T* = 0		*T* = 30 days		*T* = 60 days	
			
	Yeasts and filamentous fungi	LAB	Yeasts and filamentous fungi	LAB	Yeasts and filamentous fungi	LAB
UC	5.63 ± 0.21	7.09 ± 0.09	4.14 ± 0.81	6.00 ± 0.01	5.59 ± 0.16	6.91 ± 1.13
S	5.63 ± 0.21	7.09 ± 0.09	3.30 ± 0.43	6.15 ± 0.21	5.37 ± 0.58	7.32 ± 0.58
E	5.63 ± 0.21	7.09 ± 0.09	5.14 ± 0.68	6.30 ± 0.01^∗^	5.84 ± 0.24	6.49 ± 0.44
SDB	5.63 ± 0.21	7.09 ± 0.09	<3^∗^	8.78 ± 0.11^∗^	<1^∗^	7.46 ± 0.35
SDB-E	5.63 ± 0.21	7.09 ± 0.09	<3^∗^	8.77 ± 0.01^∗^	<1^∗^	7.76 ± 0.04


*^∗^Values within the same time differ significantly with respect to UC group (*p* < 0.05). UC, untreated control; S, substrate; E, enzyme; SDB, spray dried bacteria; SDB-E, spray dried bacteria + enzyme.*

The content of glucose (time = 0) was significantly higher (*p* < 0.05) in samples where the enzyme was added (1.50 ± 0.04, 1.30 ± 0.04; 0.48 ± 0.09; 0.38 ± 0.08, and 0.47 ± 0.06 mg/mL for E, SDB-E, UC, S, and SDB, respectively) indicating a possible fast action of the enzyme liberating simple sugars for the fermentation.

The content of acetic acid was significantly higher (*p* < 0.05) in SDB and SDB-E samples after 30 (data not shown) and 60 days of fermentation compared to UC (Table 6), indicating the production of this acid by the added heterofermentative bacteria. After 30 days of fermentation, no differences in DM content (g/100 g forage) and pH values were observed. However, the content of N_NH3_/N_T_ and CP was significantly higher (*p* < 0.05) in SDB-E micro-silos compared to UC (data not shown).

**Table 6:** Chemical analyses of micro-silos (mean ± SD) after 60 days of storage at 25°C.

Sample	Acetic acid (mg/mL)	N_NH3_/N_T_ (%)	Ash (%)	CP (%)	NDF (%)	ADF (%)	N_ADIN_/N_T_ (%)	ME (Mcal/kg DM)
UC	0.64 ± 0.01^a^	6.13 ± 0.32^a^	4.10 ± 0.50^a^	8.03 ± 0.31^a^	24.17 ± 5.15^a^	13.67 ± 2.15^a^	3.67 ± 1.04^a^	2.82 ± 0.05^a^
S	0.78 ± 0.02^a^	6.70 ± 0.62^a^	4.90 ± 0.36^b^	8.13 ± 0.45^a^	27.17 ± 1.81^a^	15.80 ± 1.28^a^	3.30 ± 0.62^a^	2.77 ± 0.03^a^
E	1.08 ± 0.01^b^	7.50 ± 1.05^a^	4.77 ± 0.38^a^	8.70 ± 0.53^a^	17.67 ± 3.61^b^	9.97 ± 2.46^b^	2.60 ± 1.42^a^	2.91 ± 0.06^b^
SDB	1.29 ± 0.19^b^	7.20 ± 0.95^a^	5.00 ± 0.44^b^	8.50 ± 0.35^a^	23.50 ± 2.92^a^	12.90 ± 1.31^a^	2.87 ± 0.67^a^	2.84 ± 0.04^a^
SDB-E	1.92 ± 0.22^c^	8.90 ± 0.17^b^	5.53 ± 0.15^b^	8.83 ± 0.38^b^	18.63 ± 1.21^a^	10.57 ± 1.44^a^	1.47 ± 0.55^b^	2.90 ± 0.03^a^


*^*a,b*^Values with different superscripts differ significantly (*p* < 0.05). UC, untreated control; S, substrate; E, enzyme; SDB, spray dried bacteria; SDB-E, spray dried bacteria + enzyme. Values referred to dry basis.*

Chemical analyses after 60 d of fermentation are shown in Table 6. No differences were found for DM content among samples (data not shown). In SDB-E micro-silos the content of N_NH3_/N_T_ and CP was significantly higher (*p* < 0.05), whereas that of N_ADIN_/N_T_ was significantly lower (*p* < 0.05) compared to the other groups. No differences were observed for NDF, ADF, and ME concentrations between samples, except for E micro-silos. Ash content was significantly higher (*p* < 0.05) in S, SDB, and SDB-E micro-silos compared to UC.

### Bucket Silos and Aerobic Stability Measurement

After 50 days of fermentation, the pH values were 3.66 ± 0.02 and 3.73 ± 0.02 for UC and SDB-E bucket silos, respectively. Total number of viable LAB was significantly higher (*p* < 0.05) in SDB-E than in UC samples (8.49 ± 0.22 and 7.11 ± 0.62 log_10_ CFU/g, respectively) and a significantly lower cell load of yeast and filamentous fungi was found in inoculated silos compared with controls (2.36 ± 1.92 and 6.51 ± 0.12, respectively). This phenomenon was also checked visually when the bucket silos were opened.

**FIGURE 4:**
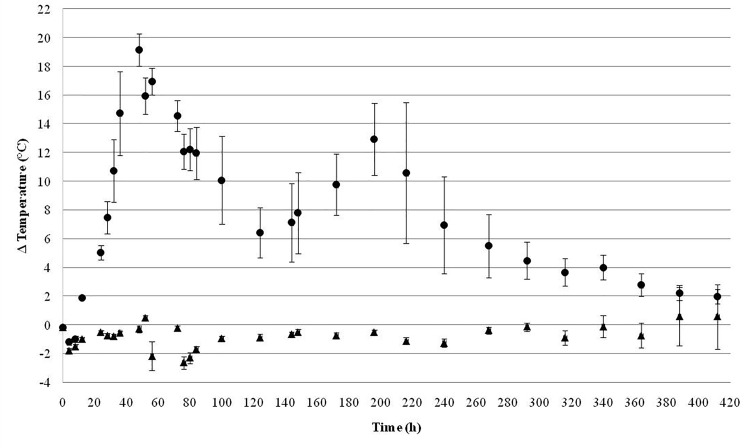
Aerobic stability of silages during time expressed as Δ temperatures (°C): the ambient temperature was subtracted from the temperature measured inside each silo (T_silo_–T_amb_). UC (●); SDB-E (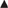). Mean values ± SD are shown.

Figure 4 shows the temperature (T_silo_-T_ambient_) of the silos during 17.5 days (420 h). The aerobic stability was significantly improved (*p* < 0.05) in SDB-E silos compared with controls since an increase of about 5°C was observed in UC samples after 24 h. On the contrary, the temperature of SDB-E silos remained stable for more than 15 days (360 h). Moreover, two maximum temperature picks were detected after 2 (48 h) and 8.16 days (196 h) in UC silos.

## Discussion

Plants are a natural habitat for certain species of LAB since these bacteria can be found on their surfaces. Even though the role of LAB on plants is not known, it is believed that they have a protective effect against pathogenic microorganisms by producing antagonistic compounds. After harvesting and compressing the plant material, *Enterococcus faecalis* and *Leuconostoc mesenteroides* usually initiate the fermentation. These are, in turn, replaced by more acid-tolerant species such as *L. brevis*, *L. plantarum*, and *L. buchneri* (Holzer et al., 2003). LAB species identified in our work correspond to those commonly found in silages (Dunière et al., 2013; Muck, 2013; Santos et al., 2013). Out of 22 isolates, 5 were obtained from silages harvested in Esperanza (from which 3 were selected for further studies), and 17 were obtained from silages harvested in Recreo (from which 8 were selected for further characterization). The criteria that were taken into account to select preliminary different isolates were: silage origin, macroscopic characteristics of the colonies grown in different media, gas production and cellular morphology (examined by phase-contrast microscopy, 1000x). Most of the strains were isolated after 7 days of natural fermentation of maize; *L. buchneri* Ls141 was isolated after 14 days and *L. amylovorus* strains were isolated after 14 or 21 days of fermentation (Table 1).

Some published data suggest that *Pediococcus* species would be responsible for starting the acidification process in silage when the growth of *Lactobacillus* strains is not favored because of the high pH value of the forage (Muck, 2013).

A successful application of inoculants in silage is based on the compatibility between the plant and the microorganisms used. This compatibility can be assessed by the ability of the microorganisms to use carbohydrates present in the forage and to produce metabolites of interest, primarily in the preservation of silage (e.g., acetic and lactic acids) (Ávila et al., 2014). In our study, *L. buchneri* Ls141 showed the highest growth ability in VBM after 24 and 48 h of incubation at 34 °C and both *L. plantarum* strains (Ls71 and Hv75) produced the largest amounts of lactic acid. *L. plantarum* is a homofermentative LAB when glucose is not a limiting factor, otherwise, it is a facultatively heterofermentative strain. This fact could explain the production of acetic acid by these strains.

Three strains were selected for further characterization and to study their performance as a spray dried culture in maize micro and bucket silos. Selected strains were *L. plantarum* Ls71 since it showed a high growth rate in MRS broth at 34°C and a high capability to produce lactic acid in VBM; *P. acidilactici* Ls72 because it was the most heat-resistant strain and *L. buchneri* Ls141 which showed good growth ability in VBM and it is a heterofermentative LAB that produces acetic acid. Moreover, these LAB species are normally found in commercial inoculants.

Regarding the capacity of the selected strains able to grow in co-culture in MRS broth and VBM, satisfactory results were obtained in MRS; nevertheless, inhibition was detected for *P. acidilactici* Ls72 when cultured together with *L. buchneri* Ls141 in VBM. Although it would appear that *L. buchneri* Ls141 inhibits, *in vitro*, the growth of *P. acidilactici* Ls72, this does not imply that the same fact would happen in silage, which is a more complex environment.

Important challenges to overcome by a strain for its industrial use are the stressors found during manufacture and storage. The three selected strains tolerated satisfactorily both spray and freeze-drying. *P. acidilactici* Ls72 was the most heat resistant strain and showed the highest resistance to spray and freeze-drying. While freeze-drying is most commonly used, other drying processes for microorganism preservation have been tested. Spray drying is a low-cost alternative to freeze-drying (only 12–20% of the cost of freeze-drying) and allows the production of large amounts of dried cells in a continuous process. The feasibility to apply spray-drying to LAB will depend on the technological conditions applied (Peralta et al., 2017; Uang et al., 2017; Reale et al., 2019). To the best of our knowledge, there are no spray dried silage inoculants in the market so far. Most of them are commercialized as freeze-dried powders or as water-soluble concentrates for which a strict cold chain is needed to warrant high cell viability.

In this study, the performance of three selected SD LAB was evaluated in maize micro and bucket-silos. Moreover, it was checked that the matrix used to spray dry the cells had no influence on the fermentation process. The most common measurements used for evaluating silage fermentation are pH, the concentration of organic acids, alcohols, NH_3_/N and microbial populations (Kung et al., 2018). Even though after 4 days of fermentation at 25°C the pH values of all samples were lower than 3.9, indicating that natural fermentation had occurred, a faster decrease was observed in both inoculated micro-silos (SDB and SDB-E). Maize silages normally have a final pH of 3.7–4.0 because it is a crop that has a low buffering capacity. The low pH from lactic acid stabilizes silage fermentation by inhibiting the growth of or killing acid-intolerant microorganisms (Kung et al., 2018). Furthermore, a significantly lower cell load of yeasts and filamentous fungi was found in inoculated micro-silos after 30 days and levels lower than 1 log_10_ CFU/g were found at the end of the fermentation period (60 days). Similar results were found in bucket silos after 50 days of fermentation.

The use of LAB as bio-preservative agents in food due to their antimicrobial activity is well documented. Among LAB, several strains *of L. plantarum* are known for their ability to produce antifungal substances (Laitila et al., 2002; Sorrentino et al., 2013). Russo et al. (2017) reported that phenyllactic acid production by *L. plantarum* has been associated with the antifungal activity against strains belonging to species of *Aspergillus*, *Penicillium*, and *Fusarium*. Therefore, the use of *L. plantarum* Ls71 in inoculated silages could have contributed to the control of filamentous fungi. On the other hand, the reduction in the number of undesirable microorganisms could also be attributed to the production of acetic acid by *L. buchneri* Ls141 which correlated with the highest concentration of acetic acid in SDB (0.90 ± 0.03 and 1.29 ± 0.19 mg/mL) and SDB-E (1.40 ± 0.33 and 1.92 ± 0.22 mg/mL) micro-silos after 30 and 60 days of fermentation, respectively. Reich and Kung (2010) reported that the cell load of yeasts was extremely low or no detectable in silages treated with the strain *L. buchneri* 40788 and higher than 4 log_10_ CFU/g in control samples. Additionally, the number of total LAB was higher in inoculated than in control silos. In a meta-analysis of the effects of *Lactobacillus buchneri* on the fermentation and aerobic stability of maize and grass and small-grain silages, Kleinschmit and Kung (2006) suggested that treatment of maize silage with the lower application rates of *L. buchneri* (< 10^5^ CFU/g of fresh forage) resulted in a 10-fold decrease in numbers of yeasts compared with the untreated silage and, the treatment with LB2 (> 10^5^ CFU/g of fresh forage) decreased the numbers of yeasts more than 100-fold.

After 30 days of fermentation chemical analyses in micro-silos showed that there was no amino acid degradation, deamination or excessive degradation of proteins. Plant and microbial proteolytic activity lead to changes in nitrogenous compounds in silages. Both E and SDB-E micro-silos showed the highest values of N_NH3_/N_T_ (6.63 ± 0.67 and 6.07 ± 0.61%, respectively), which could be due to the addition of cellulase.

After 60 days of fermentation, the pH remained below 3.90 for all samples. Values of NDF and ADF were significantly lower in E and SDB-E micro-silos compared to UC. Values of N_ADIN_/N_T_ remained below 15%, indicating that there was no production of indigestible compounds, with a significant reduction observed in SDB-E samples.

The continuous infiltration of air during the storage period of the forage in the silo facilitates the growth of aerobic microorganisms that metabolize organic matter to generate a disposable rotten material for use in animal feed. In general, acid-tolerant and lactate-assimilating yeasts initiate this process of deterioration by consuming carbohydrates and acids produced during fermentation, which generates an increase in the temperature and pH of the ensiled material (Borreani et al., 2018; Kung et al., 2018). In our work, no significantly heating was observed in SDB-E silos after 15 days (360 h), indicating a high aerobic stability. These results correlated with the lower number of yeast and filamentous fungi found in inoculated silages compared to controls.

Literature data suggest that *L. buchneri* successfully improves the aerobic stability of a variety of silages; however, the effects are strain-specific and dose-dependent (Muck et al., 2018).

## Conclusion

In this work, eleven strains belonging to six species of LAB were isolated from naturally fermented maize silage. After characterization, *L. plantarum* Ls71, *P. acidilactici* Ls72, and *L. buchneri* Ls141 were selected to be dehydrated by spray-drying and to evaluate their performances in micro and bucket-silos. Results indicate that the selected strains have the potential to be produced as a SD silage inoculant for maize ensiling since they accelerated the fermentation process, controlled the development of filamentous fungi and yeasts, improved some nutritional and chemical relevant silage quality variables and significantly enhanced aerobic stability.

## Author Contributions

PB and MB designed the study, analyzed data, and contributed to the writing of the manuscript. RP was responsible for the spray drying treatments and for the optimization of the drying process. AB performed the molecular identification of the strains. MP contributed to the experimental design of the work and to the writing of the manuscript. CB performed the HPLC analysis. JR, GV, and RM participated in the writing and revision of the manuscript and scientific discussions.

## Conflict of Interest Statement

The authors declare that the research was conducted in the absence of any commercial or financial relationships that could be construed as a potential conflict of interest.

## Footnotes

1 https://blast.ncbi.nlm.nih.gov/Blast.cgi
